# Single coronary artery originating from the right coronary sinus presented with chronic total occlusion: A case report

**DOI:** 10.1016/j.ijscr.2025.110996

**Published:** 2025-02-03

**Authors:** Xhevdet Krasniqi, Aurora Bakalli, Valon Morina, Pajazit Morina, Ramiz Qovanaj, Hajdin Çitaku

**Affiliations:** a Medical Faculty, University of Prishtina, Kosovo; b University Clinical Center of Kosova, Kosovo

**Keywords:** Single coronary artery, Coronary angiography, Coronary computed tomography angiography

## Abstract

**Introduction and importance:**

A single coronary artery (SCA) is a rare congenital anomaly in which the artery originates from either the left or right coronary sinus. Shortly after its origin, the SCA branches into the right coronary artery (RCA), the left anterior descending artery (LAD), and the left circumflex artery (LCx). When the SCA originates from the right coronary sinus, the LAD may follow one of several abnormal courses, including the posterior atrioventricular groove, retro-aortic, interarterial, intraseptal, prepulmonic, or posterior-anterior interventricular groove. Assessing the anatomical risk and the presence of concomitant coronary artery disease (CAD) in the anomalous vessel is essential for determining the appropriate treatment.

**Case presentation:**

We present a very rare case of a retro-aortic LAD course originating from SCA of the right coronary sinus in a patient admitted to our clinic for evaluation of chest pain. Coronary angiography (CA) of the right coronary system revealed a SCA. RCA was occluded, with chronic total occlusion (CTO). Coronary computed tomography angiography (CCTA) was done to specify the course of LAD, which showed a SCA and a posterior course around the aorta of LAD.

**Clinical discussion:**

CA is a valuable tool to identify and classify coronary artery anomalies (CAAs), but due to invasiveness, low spatial resolution, and lack of three-dimensional images, it has been progressively replaced by CCTA. CCTA is the gold standard for diagnosis of the CAAs that enabling three-dimensional visualization of the surrounding cardiac and non-cardiac structures identify patients with malignant CAAs. Taking into account the presence of SCA and CTO, the treatment option is recanalization of CTO or cardiac surgery if refractory angina is present.

**Conclusion:**

When the SCA originates from the right coronary sinus, identifying the abnormal course of the LAD is critical, as it may be life-threatening. Alongside CA, CCTA plays a key role in evaluating the anatomy and associated risks of this anomaly. The appropriate treatment is determined based on the presence and severity of concomitant CAD.

## Highlights

• Single coronary artery (SCA) is very rare congenital anomaly originating from the left or the right coronary sinus.
• On the origin of the SCA from the right coronary sinus, the left anterior descending artery (LAD) may have different abnormal courses (posterior atrioventricular groove, retro-aortic, interarterial, intraseptal, prepulmonic and posterior-anterior interventricular groove).
• Coronary angiography (CA) is a valuable tool to identify and classify Coronary artery anomalies (CAA), but because of invasiveness, low spatial resolution, and lack of three-dimensional images, it has been progressively replaced by coronary CT angiography (CCTA).
• Coronary computed tomography angiography (CCTA) is done to rule out a malignant course of LAD.
• Taking into account the presence of SCA and CTO, the treatment option is recanalization of CTO or cardiac surgery if refractory angina is present.

## 1 Introduction

Single coronary artery (SCA) is a subvariant of anomalous aortic origin of the coronaries [[1], [2], [3]]. Coronary artery anomalies (CAAs) have an incidence from 0.3 % to 5.6 %, whereas the prevalence is reported to be 0.3 %–2 % of the general population [4,5]. Anomalous aortic origin of coronary arteries (AAOCA) is rare with an incidence ranging from 0.1 % to 0.3 % [6]. SCA is very exceptional congenital anomaly originating from the left or the right coronary sinus and separates shortly form its origin into the right coronary artery (RCA), the left anterior descending artery (LAD) and the left circumflex artery (LCx). When the SCA originates from the right coronary sinus, the LAD may follow various abnormal courses, including the posterior atrioventricular groove, retro-aortic, interarterial, intraseptal, prepulmonic, or posterior-anterior interventricular groove [7,8].

Assessing the anatomical risks and the presence of concomitant coronary artery disease (CAD) in the anomalous vessel is essential for determining the appropriate treatment. [9].

We present a very rare case where retro-aortic LAD course originates from SCA of the right coronary sinus. The work has been reported in line with the SCARE criteria.

## 2 Case presentation

A 60-year-old Caucasian male came to our clinic for evaluation of chest pain. The pain was described as squeezing-sensation and tightness of the chest extending to the left arm. He also had breathlessness, nausea, dizziness and cold sweat. The data about personal history revealed that he suffers from high blood pressure, he is an active smoker, and has positive family history for coronary heart disease. The patient reported experiencing stable angina but had not undergone any coronary intervention. In addition to hypertension treatment, the patient was recently prescribed antiplatelet and antilipemic agents. However, neither the patient's history nor the examination indicated the presence of any malformations.

At the time of admission, patient presented with normal electrocardiogram (ECG). The levels of cardiac biomarkers were within normal range: creatine kinase-MB (CK-MB) level of 20 U/L (7–25 U/L), creatine kinase level 105 U/L (38–171 U/L), and troponin I level of 0.01 ng/mL (0-02 ng/mL). Transthoracic echocardiography revealed hypokinesis of basal and mid inferior wall, with normal ejection fraction of the left ventricle (LVEF≈50 %).

**Fig. 1:**
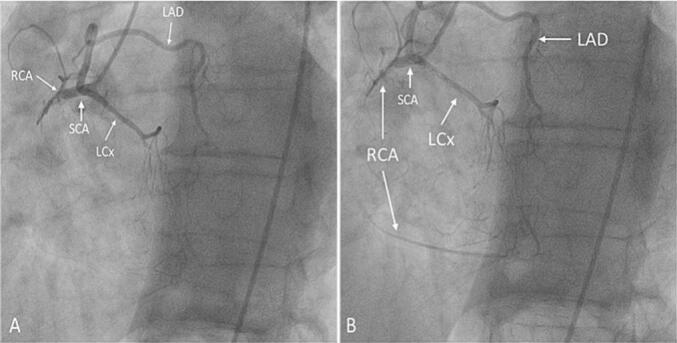
Coronary angiography revealing single coronary artery (SCA). Right coronary artery (RCA) is occluded-chronic total occlusion (CTO) (A) with retrograde filling flow from the left anterior descending (LAD) artery and the left circumflex (LCx) artery (B).

Coronary angiography was performed in the left anterior oblique caudal (LAO-caudal) projection, but it was not possible to engage the left coronary artery. Coronary angiography of the right coronary system revealed SCA. RCA, LAD and LCx branch originated from SCA where LAD has posterior course to the aorta. RCA was occluded-chronic total occlusion (CTO) with retrograde filling flow from the left anterior descending (LAD) artery and the LCx artery [Fig. 1].

**Fig. 2:**
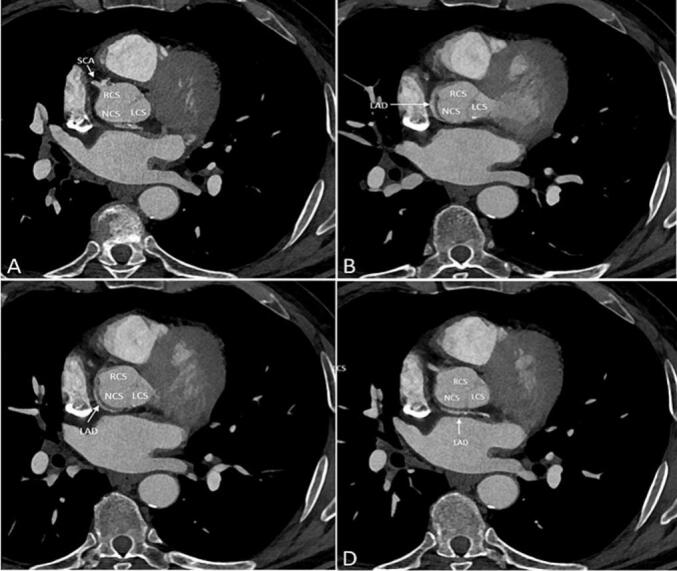
Coronary CT Angiography showing single coronary artery (SCA) (A) and a posterior course around the aorta of left anterior descending artery (LAD) (B-C).

Coronary computed tomography angiography (CCTA) was done to rule out a malignant course of LAD. CCTA confirmed the SCA and a posterior course around the aorta of the LAD [Fig. 2].

## 3 Discussion

Our case presentation presents a very rare congenital anomaly where SCA originates from the right coronary sinus. The incidence of anomalous origin of the left coronary artery from the right sinus of Valsalva (ALCA) is 0.15 %, and the right coronary artery from the left coronary sinus (ARCA) is 0.92 % [5,6]. Isolated SCA, as uncommon anomaly, is found in a very small number of cases, the incidence is 0.024 % to 0.066 % [10]. Based on the Lipton anatomical classification, SCA may be type I, II and III, with subtypes depending from the origin, the right or left coronary sinus (RI, RII, RIII, LI and LII) [11]. In the type I, the course of the SCA corresponds to the same course of normal left coronary artery (LCA) or RCA. The type II is characterized with abnormal origin, one coronary artery from proximal segment of the other coronary artery. In type III, SCA originates from the right coronary sinus where LAD and LCx are separated from the common trunk [12]. There are five possible paths based on the reperfusion territory: (1) pre-pulmonic; (2) retro-aortic; (3) trans-septal; (4) retro-cardiac; and (5) inter-arterial [[13], [14], [15]]. Myocardial ischemia in patients with CAA results from certain factors: ostial stenosis, ostial ridge, vessel spasm, intussusception, noncompliant pericommissural area, and compression of the anomalous coronary artery in the intramural or inter-arterial course [16]. Only inter-arterial course is malignant and can be followed with sudden cardiac death (SCD) [5].

Our case is type RIII, according to Lipton anatomical classification, whereas based on reperfusion territory it is characterized with retro-aortic course. Myocardial ischemia results from the chronic total occlusion (CTO) of the RCA [17].

Coronary angiography (CA) is a valuable tool to identify and classify CAAs, but due to invasiveness, low spatial resolution, and lack of three-dimensional properties, it has been progressively replaced by coronary CT angiography (CCTA) [1,18]. CCTA is the gold standard for diagnosis of the CAAs that enables three-dimensional visualization of the surrounding cardiac and non-cardiac structures, determines risk anatomy, and identifies malignant CAAs [9,19,20].

Taking into account the presence of SCA and CTO, the treatment option is recanalization of CTO or cardiac surgery if refractory angina is present [16,17,21].

The work has been based on the SCARE 2023 Criteria [22].

## 4 Conclusions

When the SCA originates from the right coronary sinus, identifying the abnormal course of the LAD is critical, as it may be life-threatening. In addition to CA, CCTA plays a key role in evaluating the anatomy and associated risks of this anomaly. The appropriate treatment is determined based on the presence and severity of concomitant CAD, recanalization of CTO or cardiac surgery.

## Author contribution

XK was the first author. XK, AB, VM, PM, RQ and HÇ prepared the final manuscript. All authors contributed to data collection and read and approved the final manuscript.

## Consent

Written informed consent was obtained from the patient for the publication of this case report and any accompanying images. A copy of the written consent is available for review by the Editor-in-Chief of this journal.

## Ethical approval

Ethical approval for this study (Ethical Committee N° 17408) was provided by the Ethical Committee of the Medical Faculty, University of Prishtina “Hasan Prishtina”, Prishtina, Kosova on 15 November 2024.

## Guarantor

Aurora Bakalli.

## Research registration number

Not applicable.

## Funding

Not applicable.

## Conflict of interest statement

The authors declare that they have no competing interests.

## Data Availability

All data from this study are included.
